# A mathematical analysis of mass transfer phenomena with chemical reaction over the flow of Sisko ferronanofluid across a permeable surface

**DOI:** 10.1038/s41598-022-27214-7

**Published:** 2023-01-07

**Authors:** K. Saritha, R. Muthusami, N. Manikandan, N. Nagaprasad, Krishnaraj Ramaswamy

**Affiliations:** 1 Department of Mathematics, P.A.College of Engineering and Technology, Pollachi, Tamil Nadu 642002 India; 2 Department of Computer Applications, Dr. Mahalingam College of Engineering and Technology, Pollachi, Tamil Nadu India; 3 Department of Mechanical Engineering, P.A. College of Engineering and Technology, Tamil Nadu, Pollachi, 642002 India; 4 Department of Mechanical Engineering, ULTRA College of Engineering and Technology, Madurai, Tamil Nadu 625 104 India; 5 entre for Excellence-Indigenous Knowledge, Innovative Technology Transfer and Entrepreneurship, DambiDollo University, Dembi Dola, Ethiopia; 6 Department of Mechanical Engineering, DambiDollo University, Dembi Dola, Ethiopia

**Keywords:** Biochemistry, Chemistry, Engineering, Materials science, Mathematics and computing

## Abstract

Mathematically study mass transfer phenomena involving chemical reactions in the flow of Sisko Ferro nanofluids through the porous surface. Three ferronano particles, manganese-zinc ferrite (Mn1/2Zn1/2Fe_2_O_4_), cobalt ferrite (CoFe_2_O_4_), and nickel–zinc ferrite (Ni–Zn Fe_2_O_4_) are considered with water (H_2_O) and ethylene glycol (C_2_H_6_O_2_) as base liquids. Appropriate resemblance transitions are used to convert the governing system of a nonlinear PDE to a linear ODE. The Runge–Kutta method, as extended by the shooting technique, is used to accomplish the reduction governing equations. The effects of various associated parameters on fluid concentration and mass transfer rate are investigated: magnetic criterion (M), Siskofluid material factor (A), Solid volume fraction (ϕ) for nanofluids, permeability parameter (Rp), Chemical reaction criterion (γ), Brownian motion factor (Nb), and Thermophoretic parameters (Nt). The current findings indicate that the diffusion proportion of Sisko Ferronanofluid Ni–Zn Fe_2_O_4_–H_2_O and CoFe_2_O_4_–H_2_O is higher than that of Ni–Zn Fe_2_O_4_–C_2_H_6_O_2_ and CoFe_2_O_4_–C_2_H_6_O_2_ respectively but it is opposite in the case of Mn–Zn ferrite. The comparison study was carried out to validate the precision of the findings.

## Introduction

A framework is made up of one or more elements, the number of which varies from system to system. Mass is also routed, reducing intensity differences within the framework. The field of mass transfer encounters some of the most unusual solutions in substance engineering. The ability to develop and operate used to make preparations for responding elements is what distinguishes a synthetic expert. Factors caused the isolation of production knowledge. One such potential seems to be highly dependent on mastery of mass transfer research. The strategies of inertia and heat transition are widely used in a variety of engineering fields, but absorption has typically been confined to bioengineering. These significant ones include fabrication systems and, more recently, the elevated airliner configuration.

Ordinary diffusion and convection have been selected for study among a number of physical processes that can move and transfer a chemical species through a system through the boundaries of the system. Mass diffusion, which happens when a gradient in species concentration develops, is comparable to heat conduction. In terms of fundamentals, mass convection and heat convection are the same; a fluid flow that carries heat can also convey a chemical species. Heat transmission and mass transfer have very similar fundamental mechanisms. It is well known that non-Newtonian fluid mechanics pose Engineers, Physicists, and Mathematicians with a special challenge. In recent years various scientists have proposed several models of non-Newtonian fluids to investigate the flow behavior of such engineering and non-engineering systems. This is because of their varying rheological characteristics. The Sisko model was commonly used by these models to model industrial and non-industrial problems.

Buongiorno's model is used to investigate the steady flow of a nanofluid of heat, thermal, and condensation mechanisms with provisional slip effects^1^. Primarily concerned with a symmetric elasticized sheet with a mixed convection MHD flow of cross fluid^2^. Fluid dynamic resilience of the Couette flow of a fluid that conducts electricity going to flow in an analogous link with a typical magnetism across a porous channel was investigated^3^. Williamson nanofluid MHD shear layer progression across a stretch sheet of the porous layer while accounting for acceleration and radiative deterioration. The goal of this model is to investigate the conditions of heat and mass transit using thermophoresis and Brownian acceleration with an approximate solution^4^. Study examines the impact of radiant heat, chemical thermolysis, and source of heat on the flow of MHD Casson fluid across a nonlinear inclined stretching surface with velocity slip in a porous medium^5^.The primary goal of this work is to quantitatively alleviate a Cross nanofluid surging across a perpetually broadening horizontal cylinder under non-Newtonian uncertain conditions^6^. Diffusion and heat conduction of a designed nanomaterial model is possible within existence besides a binary decomposition solution, radiation, as well as nanofluid, assuming Brownian motion and thermophoresis occurrences. Deals the magnetohydrodynamics of a Casson nanofluid flowing toward a stretched sheet. Additionally, the interaction between the Arrhenius activation energy, nonlinear radiation, and the current mass flow theory is examined^7^. Describes how Brownian motion and thermophoresis affect the flow of non-Newtonian power-law nanofluids in an expanding surface at a mixed convective-magneto-hydrodynamic interface^8^. In rectangular tubes with protrusions, the properties of the non-Newtonian stream and heat transport are quantitatively explored^9^. We investigate the MHD fluid Casson flow using a temperature diffusion heat absorber. It is investigated how synthetic reactivity and Joule heating function when thermal radiation passes through a porous sloped extended sheet of MHD Casson nanofluid^10^. When thermal radiation occurs through a porous sloped extended sheet of MHD Casson nanofluid, the role that chemical reaction and Joule heating play is explored in work, ramping power electromagnetic fluids stream is used thru a sloped parabolic shaped Riga area^11,12^. The Riga surface's electrically conducting viscoelastic fluid characteristics are also carefully examined, with an emphasis on time-dependent density and temperature changes.

Sisko fluid models successfully predict flow behaviour in the power law region, upper Newtonian region, and higher peak intensities.The model first learned oil flow but was later discovered to also exhibit flow behavior such as concrete glue. The manufactured products are a combination of water-based paints, flat oils, and concrete slurries. Studied the Sisko fluid boundary layer flow with entropy generation^13^. The analysis is performed on the unsteady MHD thin-film flow of Sisko Fluids when thermal radiation is applied to the expansive surface quantitative studies have been done on how heat and mass transfer affect non-Newtonian Sisko fluid flow in crooked, spongy, sloping tubes^14,15^. The movement of nanomaterials by radially contracting or extending the surface with zero mass flux, as well as the impact of magnetic fields on the movement of the Sisko liquids, were explored^16^.

Colloidal liquids called ferrofluids contain nanoscale or ferromagnetic particles floating in a carrier fluid. Ferrofluids are a unique class of nanofluids created by dispersing iron-containing nanoparticles into regular base liquids in a colloidal suspension. These liquids are extraordinary materials with fluid, magnetic, and strong thermal conductivity qualities. The Ferrofluid's strength is in its ability to control fluid flow.

In the domains of nautical technology, aviation, bioliquids, and medicinal and fibre manufacturing, it has a wide range of applications. To enhance microscale mass transfer, a dilute Ferrofluid is employed along with a non-uniform magnetic flux major objective of the work was the experimental investigation of the fluid and thermally transport characteristics of a ferrofluid depending on turbine oil and iron nanomaterial, magnetic field application^17,18^. The flow of MHD nanofluids made of water and kerosene over a porous surface with a predetermined heat flux is examined^19^.

An attenuated ferrofluid and a non-uniform magnetic flux are used to improve atomic level absorption^20^. An exploratory analysis of the motion and radiative transport properties of a ferrofluid relying on turbine oil and a ferrous nanomaterial, magnetic system, was the main objective of the work^21^.

As far as the author is aware, the current study is examining the mass transfer on flow through a permeable surface saturated with Sisko ferro nanofluid. With the required surface mass flux, the altered governing equations have a solution. For an explanation of the concentration profiles, numerical findings are shown on graphs. Table entries were used to display the excess surface concentration gradient connected to the mass flux distributions (Nt) for special values of the Siskofluid variation (A), magnetic factor (M), permeability criterion (Rp), nanoparticle volume fraction (ϕ), Brownian motion criterion (Nb), and thermophoresis parameter (Nt).

## Formulation of the problem

**Figure 1:**
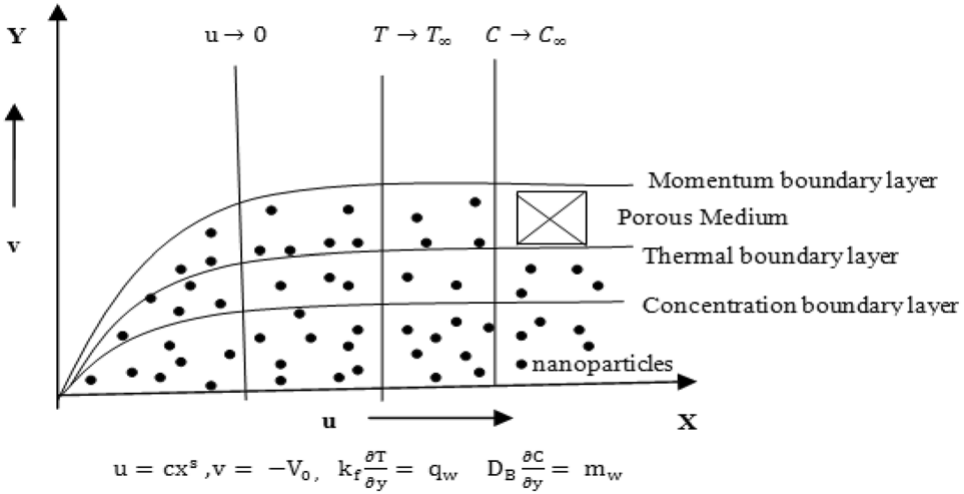
Physical illustration and coordinate of the system.

The impact of chemical reaction mass transfer in Sisko Ferronanofluid through a linearly expanding permeable surface is examined in this paper while prescribed mass flux conditions are present. The Ferrofluid model combines Brownian motion and thermophoresis to describe non-Newtonian fluid dynamics. Three Ferroparticles nickel zinc ferrite (Ni–Zn Fe_2_O_4_), cobalt ferrite (CoFe_2_O_4_), manganese zinc ferrite (Mn1/2Zn1/2Fe_2_O_4_), and two base fluids ethylene glycol and water are considered. The Ferronanofluids' physical, and thermal characteristics are shown in Table 1. The graphical abstract of the proposed research work is shown in Fig. 1. Here, the Sisko Ferro nanofluid's governing flow equations are written in the system of Cartesian coordinates. For the isotropic incompressible flow of a Sisko fluid, the rheological equation of state is given by^22^.

$$ \uptau = - {\text{PI}} + {\text{S}},{\text{ S}} = \left\lfloor {a + b\left| {\sqrt {\frac{1}{2}tr\left( {A_{1}^{2} } \right)} } \right|^{n - 1} } \right\rfloor A_{1} $$

**Table 1:** Water and ferronanoparticlesthermo physical properties.

	Density ρ (kg/m^3^)	Specific heat C_p_ (J/kg K)	Thermal conductivity K(W/mK)	Prandtl number Pr
Water (H_2_O)	997.1	4179	0.613	6.2
Ethylene glycol (C_2_H_6_O_2_)	1115	2382	0.258	204
Nickel zinc ferrite (Ni–Zn Fe_2_O_4_)	4800	710	6.3	–
Manganese zinc ferrite (Mn1/2Zn1/2Fe_2_O_4_)	4900	800	5	–
Cobalt ferrite (Co Fe_2_O_4_)	4907	700	3.7	–

The problem's governing hypothesis under the aforementioned conditions^23^.

1 $$ \frac{\partial u}{{\partial x}} + \frac{\partial v}{{\partial y}} = 0 $$

2 $$ {\text{u}}\frac{{\partial {\text{u}}}}{{\partial {\text{x}}}} + {\text{ v}}\frac{{\partial {\text{u}}}}{{\partial {\text{y}}}}{ } = { }\frac{{\text{a}}}{{{\uprho }_{{{\text{nf}}}} }}\frac{{\partial^{2} {\text{u}}}}{{\partial {\text{y}}^{2} }} - \frac{{\text{b}}}{{{\uprho }_{{{\text{nf}}}} }}\frac{\partial }{{\partial {\text{y}}}}\left( { - \frac{{\partial {\text{u}}}}{{\partial {\text{y}}}}} \right)^{{\text{n}}} - { }\frac{{{\sigma B}_{{0{\text{ u}}}}^{2} }}{{{\uprho }_{{{\text{nf}}}} }} - \frac{{{\upupsilon }_{{{\text{nf}}}} }}{{{\text{K}}_{{\text{p}}} }}{\text{u}} $$

3 $$ \begin{gathered} \frac{\partial T}{{\partial x}} + v\frac{\partial T}{{\partial y}} = \alpha_{nf} \frac{{\partial^{2} T}}{{\partial y^{2} }} - \frac{1}{{\left( {\rho C_{P} } \right)_{nf} }}\frac{{\partial q_{r} }}{\partial y} - Q^{\prime } \frac{\partial T}{{\partial y}} + \frac{a}{{\left( {\rho C_{P} } \right)_{nf} }}\left( {\frac{\partial u}{{\partial y}}} \right)^{2} + \frac{b}{{\left( {\rho C_{P} } \right)_{nf} }}\left( { - \frac{\partial u}{{\partial y}}} \right)^{n + 1} + \hfill \\ \frac{{\left( {\rho C_{p} } \right)_{p} }}{{\left( {\rho C_{p} } \right)_{f} }}\left[ {D_{B} \frac{\partial C}{{\partial y}}\frac{\partial T}{{\partial y}} + \frac{{D_{T} }}{{T_{\infty } }}\left( {\frac{\partial T}{{\partial y}}} \right)^{2} } \right] \hfill \\ \end{gathered} $$

4 $$ {\text{u}}\frac{{\partial {\text{C}}}}{{\partial {\text{x}}}} + {\text{ v}}\frac{{\partial {\text{C}}}}{{\partial {\text{y}}}}{ } = {\text{ D}}_{{\text{B}}} \frac{{\partial^{2} {\text{C}}}}{{\partial {\text{y}}^{2} }} + \frac{{{\text{D}}_{{\text{T}}} }}{{{\text{T}}_{\infty } }}\frac{{\partial^{2} {\text{T}}}}{{\partial {\text{y}}^{2} }}{ } - {\text{ k}}_{1} \left[ {{\text{C}} - {\text{C}}_{\infty } } \right] $$

The conservation equations governing the flows

5 $$ \begin{gathered} u\left( {x,y} \right) = U = cx^{s} \hfill \\ v\left( {x,y} \right) = - V_{0} \hfill \\ k_{f} \frac{\partial T}{{\partial y}} = q_{w } = E_{0} \left( \frac{x}{L} \right)^{m} at\quad y \, = \, 0 \hfill \\ D_{B} \frac{\partial C}{{\partial y}} = m_{w } = E_{1} \left( \frac{x}{L} \right)^{n} at\quad y = 0 \hfill \\ u = 0{ },{ }T{ } \to T_{{\infty { }}} ,{ }C{ } \to C_{{\infty { }}} \;as{ }y \to \infty { } \hfill \\ \end{gathered} $$

In which L ‘is characteristic length, ‘s’ is power-law velocity index,‘c’ is constant and ${ }^{\prime } {\text{m}}_{{\text{w }}}^{\prime }$ is surface mass flux.

$$ \mu_{nf} = \frac{{\mu_{f} }}{{\left( {1 - \phi } \right)^{2.5} }},\upsilon_{nf} = \frac{{\mu_{nf} }}{{\rho_{nf} }},\rho_{nf} = \left( {1 - \phi } \right)\rho_{f} + \phi \rho_{p} $$

The following similar transformations (Malik^24^).

${\text{Q}}^{^{\prime}} = - {\text{d Q f}}\left( {\upeta } \right)$ Seems to be the parametric proportion of a thermal regression model.

$$ d = UR_{{e_{b} }}^{{\frac{ - 1}{{n + 1}}}} ,\psi = UxR_{{e_{b} }}^{{\frac{ - 1}{{n + 1}}}} f\left( \eta \right),\eta = \frac{y}{x}R_{{e_{b} }}^{{\frac{1}{n + 1}}} , $$

$$ u = f^{^{\prime}} \left( \eta \right)U $$

$$ v = - U R_{{e_{b} }}^{{\frac{ - 1}{{n + 1}}}} \frac{1}{n + 1}\left[ {\left\{ {s\left( {2n - 1} \right) + 1} \right\}f\left( \eta \right) + \left\{ {s\left( {2 - n} \right) - 1} \right\}\eta f^{^{\prime}} \left( \eta \right)} \right] $$

$$ T - T_{\infty } = \frac{{E_{0} }}{{k_{f} }}\left( \frac{x}{L} \right)^{m} x R_{{e_{b} }}^{{\frac{ - 1}{{n + 1}}}} g\left( \eta \right) $$

6 $$ C - C_{\infty } = \frac{{E_{1} }}{{D_{B} }}\left( \frac{x}{L} \right)^{n} x R_{{e_{b} }}^{{\frac{ - 1}{{n + 1}}}} h\left( \eta \right) $$

Transformed equations of (2), (3), and (4)

7 $$ Af^{\prime \prime \prime } + n\mathop {\left( { - f^{\prime } } \right)}\nolimits^{n - 1} f^{\prime \prime \prime } + \mathop \varphi \nolimits_{1} \left( {\frac{s(2n - 1) + 1}{{n + 1}}ff^{\prime \prime } - sf^{\prime 2} } \right) - \left( {M + \frac{1}{{R_{p} \phi_{2} }}} \right)f^{\prime } = 0{\kern 1pt} $$

8 $$ g^{\prime \prime } + D\left( {\frac{s(2n - 1) + 1}{{n + 1}} + Q} \right)fg^{\prime } - (m + 1)Df^{\prime } g + DN_{b} g^{\prime } h^{\prime } + N_{t} Dg^{\prime \prime 2} = - \frac{{E_{c} D}}{{\phi_{3} }}\left( {A\mathop {\left( {f^{\prime \prime } } \right)}\nolimits^{2} + \mathop {\left( { - f^{\prime \prime } } \right)}\nolimits^{n + 1} } \right){\kern 1pt} $$

9 $$ h^{\prime \prime } + LeP_{r} \frac{s(2n - 1) + 1}{{n + 1}}f \, h^{\prime } - (m + 1)LeP_{r} \, f^{\prime } h - \gamma LeP_{r} h + \frac{{N_{t} }}{{N_{b} }}\mathop g\nolimits^{\prime \prime } = 0 $$

As for model parameters

$$ \left. {\begin{array}{*{20}c} {f^{\prime } \left( \eta \right) = 1, \quad f\left( \eta \right) = \lambda } \\ {g^{\prime } \left( \eta \right) = - 1, \quad h^{\prime } \left( \eta \right) = - 1, } \\ \end{array} } \right\}\quad at\quad \eta = 0 $$

10 $$ f^{\prime } \left( \eta \right) = 0 , \, g\left( \eta \right) = 0, h\left( \eta \right) = 0\quad as\quad \eta \to \infty $$

where $\phi_{1} = 1 - \phi + { }\phi \frac{{{\uprho }_{{\text{s}}} }}{{{\uprho }_{{\text{f}}} }}$, $\phi_{2} = { }\left( {1 - \phi } \right)^{2.5}$, $A = \frac{{R_{{e_{b} }}^{{\frac{2}{n + 1}}} }}{{R_{{e_{a} }} }}$ is Sisko fluid parameter $R_{{e_{a} }} = \frac{{\rho_{f} x U}}{a}$ and $R_{{e_{b} }} = \frac{{\rho_{f } x U^{2 - n} }}{b}$ are Reynolds number, $\lambda = \frac{{V_{0} }}{{U R_{{e_{b} }}^{{\frac{ - 1}{{n + 1}}}} }}$ is a suction parameter, $R_{p} = \frac{{K_{P} U}}{{\nu_{f} x}}$ is permeability parameter, $M = \frac{{\sigma B_{0 x}^{2} }}{{\rho_{f U} }}$ is a magnetic parameter, $P_{r} = \frac{{x U R_{{e_{b} }}^{{\frac{ - 2}{{n + 1}}}} }}{{\alpha_{f} }}$ is Prandtl number, $\gamma = {\kern 1pt} \frac{{k_{1} x}}{U}$ (chemical reaction parameter), $\mathop L\nolimits_{e} = \;{\kern 1pt} \frac{{\mathop \alpha \nolimits_{f} }}{{\mathop D\nolimits_{B} }}$ (Lewis number),$N_{b} = \frac{{\left( {\rho C_{p} } \right)_{p} }}{{\left( {\rho C_{p} } \right)_{f} }} \frac{{D_{B } \left( {C_{f } - C_{\infty } } \right)}}{{\alpha_{f} }}$ is Brownian motion parameter, $N_{t} = \frac{{\left( {\rho C_{p} } \right)_{p} }}{{\left( {\rho C_{p} } \right)_{f} }} \frac{{D_{T } \left( {T_{f } - T_{\infty } } \right)}}{{T_{\infty } \alpha_{f} }}$ is thermophoresis parameter.

The physical quantity Sherwood number is defined from the relation

11 $$ N_{{u_{x} }} = \frac{{xm_{w} }}{{D_{B} \left( {C_{f } - C_{\infty } } \right)}} at \quad y = 0 $$

Using Eqs. (4), (5), and (9), the dimensionless Sherwood number can be written as

12 $$ {\text{R}}_{{{\text{e}}_{{\text{b}}} }}^{{\frac{ - 1}{{{\text{n}} + 1}}}} {\text{S}}_{{{\text{h}}_{{\text{x}}} }} = \frac{1}{{{\text{h}}\left( 0 \right)}} $$

### Numerical methods

In this study, we investigate the flow of MHD Sisko ferronano fluids under the influence of chemical reactions and specific mass flow rates. The Runge–Kutta method extended by the shooting technique is used for solving Eqs. (7) to (9) for a range of values ​​of some specific parameters. To select a good initial approximation, the models have evolved into first-order nonlinear equations^25^.

Define a new set of variables as

13 $$ f\left( \eta \right) = x_{1} ,\;f^{\prime } \left( \eta \right) = x_{2} ,\;f^{\prime \prime } \left( \eta \right) = x_{3} ,\;g\left( \eta \right) = x_{4} ,\;g^{\prime } \left( \eta \right) = x_{5} ,\;h\left( \eta \right) = x_{6} ,\;h^{\prime } \left( \eta \right) = x_{7} $$

Equation (13) is imposed on Eqs. (7)–(9), resulting in a method of seven ordinary differential equations as

14 $$ \frac{{dx_{1} }}{d\eta } = x_{2} $$

15 $$ \frac{{dx_{2} }}{d\eta } = x_{3} $$

16 $$ \frac{{dx_{3} }}{d\eta } = \frac{{\phi_{1} \left( {sx_{2}^{2} - \frac{s(2n - 1) + 1}{{n + 1}}x_{1} x_{3} } \right) + \left( {M + \frac{1}{{\mathop R\nolimits_{p} \varphi_{2} }}} \right)x_{2} }}{{A + n\left( { - x_{3} } \right)^{n - 1} }} $$

17 $$ \frac{{dx_{4} }}{d\eta } = x_{5} $$

18 $$ \begin{gathered} \frac{{dx_{5} }}{d\eta } = \frac{{ - E_{c} D}}{{\mathop \varphi \nolimits_{3} }}\left( {A\mathop {\left( {x_{3} } \right)}\nolimits^{2} + \mathop {\left( { - x_{3} } \right)}\nolimits^{n + 1} } \right) - \left( {\frac{s(2n - 1) + 1}{{n + 1}} + Q} \right)Dx_{1} x_{5} + \hfill \\ (m + 1)Dx_{2} x_{4} - DN_{b} x_{5} x_{7} - x_{5}^{2} DN_{t} \hfill \\ \end{gathered} $$

19 $$ \frac{{\;d\mathop x\nolimits_{6} }}{d\eta }\; = \;\mathop x\nolimits_{7} $$

20 $$ \begin{gathered} \frac{{dx_{7} }}{d\eta } = - \frac{s(2n - 1) + 1}{{n + 1}}L_{e} P_{r} x_{1} x_{7} + (m + 1)L_{e} P_{r} x_{2} x_{6} + \gamma L_{e} P_{r} x_{6} - \hfill \\ \frac{{N_{t} }}{{N_{b} }}\frac{{d_{x5} }}{d\eta } \hfill \\ \end{gathered} $$

21 $$ x_{1} (0) = \lambda ,\;x_{2} (0) = 1,\;x_{3} (0) = \lambda_{1} ,\;x_{4} (0) = \lambda_{2} ,\;x_{5} (0) = - 1,\;x_{6} (0) = \;\lambda_{3} ,x_{7} (0) = - 1 $$

To solve Eqs. (7) and (9) using (10) as the initial value problem, no initial values ​​for fʺ(0), g(0) and h(0) are given. Then use the shooting estimation technique to change the attributes ​​of fʺ(0) and g(0) = 0 and assess the observed attributes ​​of f′ and g with given parameters ${\text{f}}^{\prime } \left( {{\upeta }_{\infty } } \right) = 0{ }$ and ${\text{g}}\left( {{\upeta }_{\infty } } \right) = 0.$ This process continues until the findings are accurate and meet the convergence parameter^24,26–30^.

## Results and discussion

This research looks at the fluid of MHD Sisko's ferro nanofluids through a porous stretched surface using chemical reactions. The aforementioned numerical scheme is used for the conceptual evaluation of different flow parameters. The effects of parameters such as the magnetic parameter (M), the material parameter (A) for Siskofluid, the solid volume fraction (ϕ) for nanofluids, the permeability parameter (Rp), the thermophoretic parameters (Nt), the Brownian motion factor (Nb), and the chemical reaction criterion (γ) on concentration and Sherwood quantity are shown graphically and tabulated^31^.

A comparison study was conducted using Table 2 to validate the accuracy of results obtained from previously published data. In a few specific cases, the current h(0) findings have been found to be in good accordance to previously published work.

**Table 2:** h (0) Comparison (${\uplambda }$ = 1.5, Rp = 100, Sc = 0.62, *ϕ* = 0, γ = 0).

M	Anjali Devi et al.^32^ (Analytic method)	Present work (Numerical method)
0	0.676774	0.67677428
1	0.697755	0.69775508
4	0.737861	0.73786087
9	0.776667	0.77666685
16	0.809680	0.80967977

**Figure 2:**
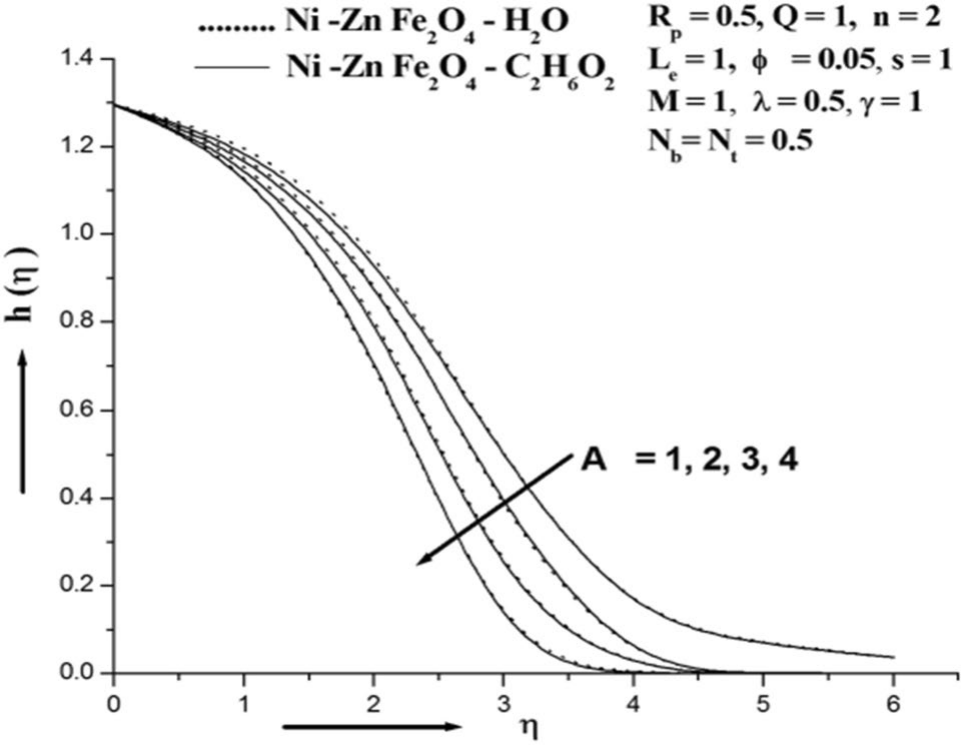
Illustration of concentration for various A.

Figure 2 depicts the characterization of the material parameter Siskofluid Parameter A in a diffusion coefficient. The graph illustrates that the consistency decreases as the value of A increases. A measure of the viscosity of a fluid that slows it down and reduces the concentration layer thickness.

**Figure 3:**
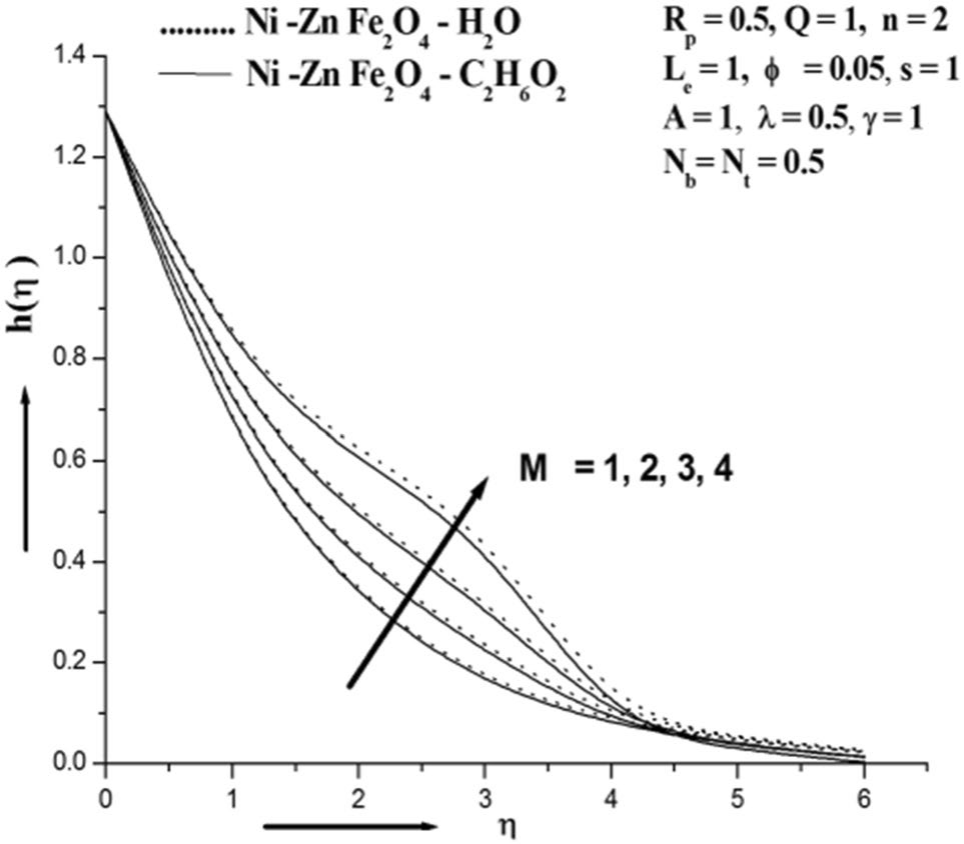
Illustration of concentration for various M.

**Figure 4:**
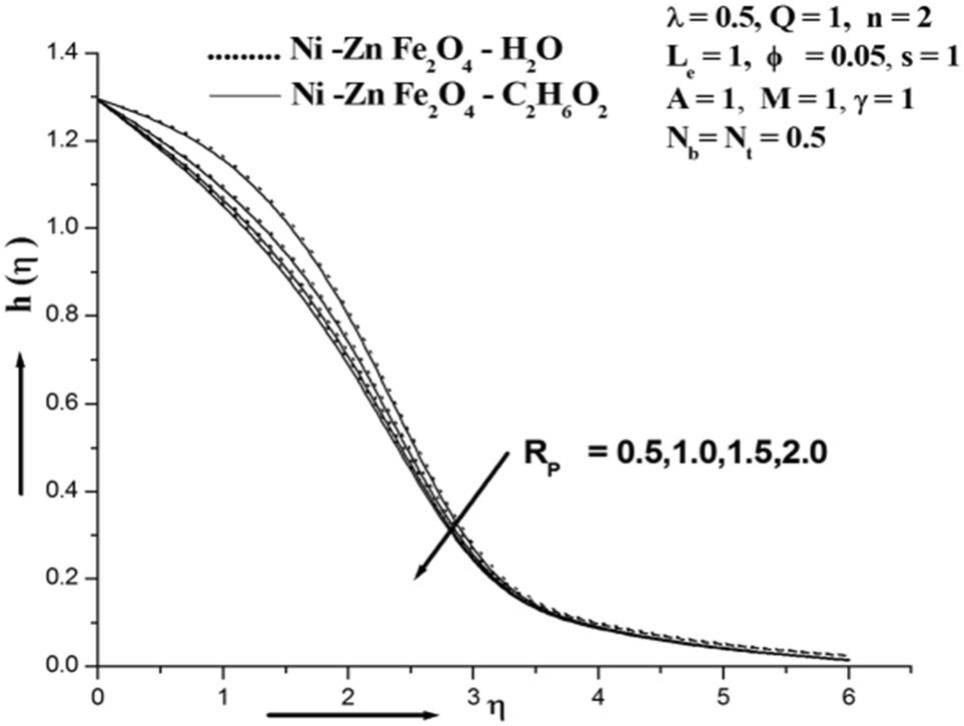
Illustration of concentration for various R_p_.

**Figure 5:**
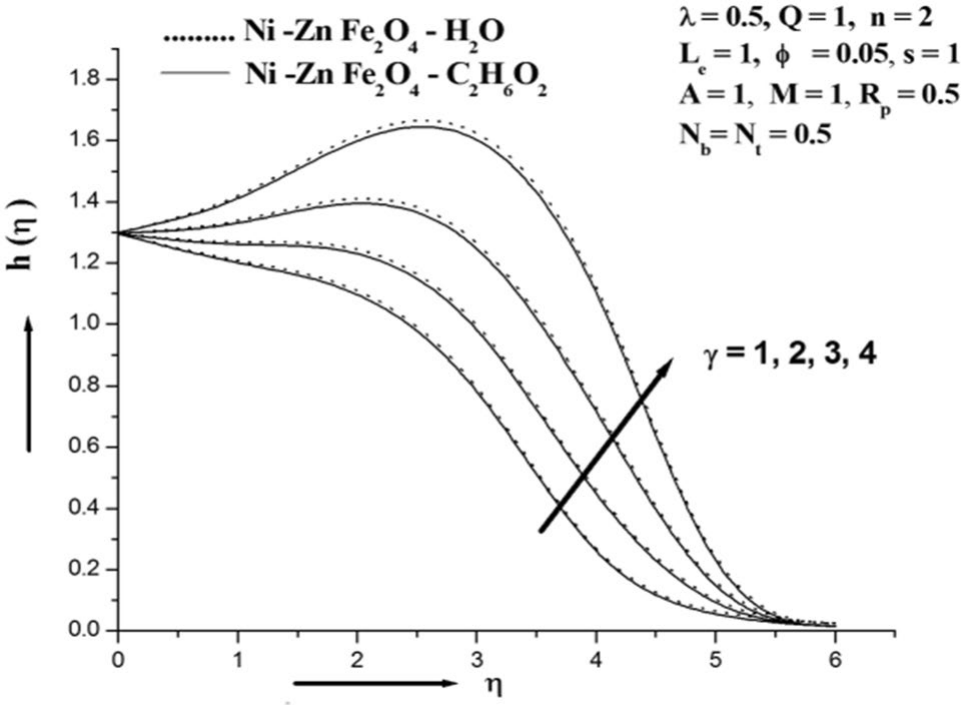
Illustration of concentration for various γ.

Figure 3 illustrates how the magnetic field affects Concentration. It can be demonstrated that the concentration rises as the value of M rises. The Lorentz force is a resistive force that results from the transverse magnetic field's actions on an electrically conducting fluid. This force aids in slowing the fluid's velocity and improving its concentration profile. Given that the magnetic field slows the mixed convection flow, this discovery qualitatively fits forecasts. A driving force that slows down the fluid's motion and thickens its boundary layer. The magnetic field causes high collisions between the ferro nanoparticles in the inter-particles, which raises the concentration. Figure 4 illustrates how the permeability parameter Rp affects the absorption profile. And is obvious further that the existence of a porous material increases the flow's constraint, which slows the fluid down. The velocity drops as a result of the impermeability parameter increasing the resistance to fluid motion. Figure 5 illustrates the chemical reaction parameter γ impact on concentration. The value of chemical reaction parameter γ is seen to be optimised when concentration is shown to grow. A rise in the reaction rate parameter causes a high collision of ferro nanoparticle inter-particles, which raises the concentration. The study's findings suggest that water-based ferro nanofluids increase concentration when compared to those based on ethylene glycol^33–37^.

**Figure 6:**
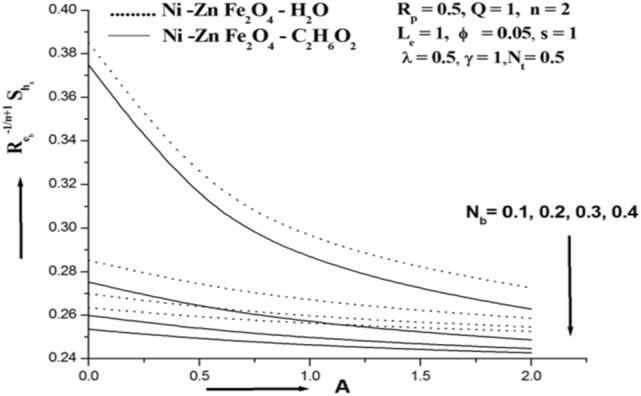
Sherwood number for various N_b_.

**Figure 7:**
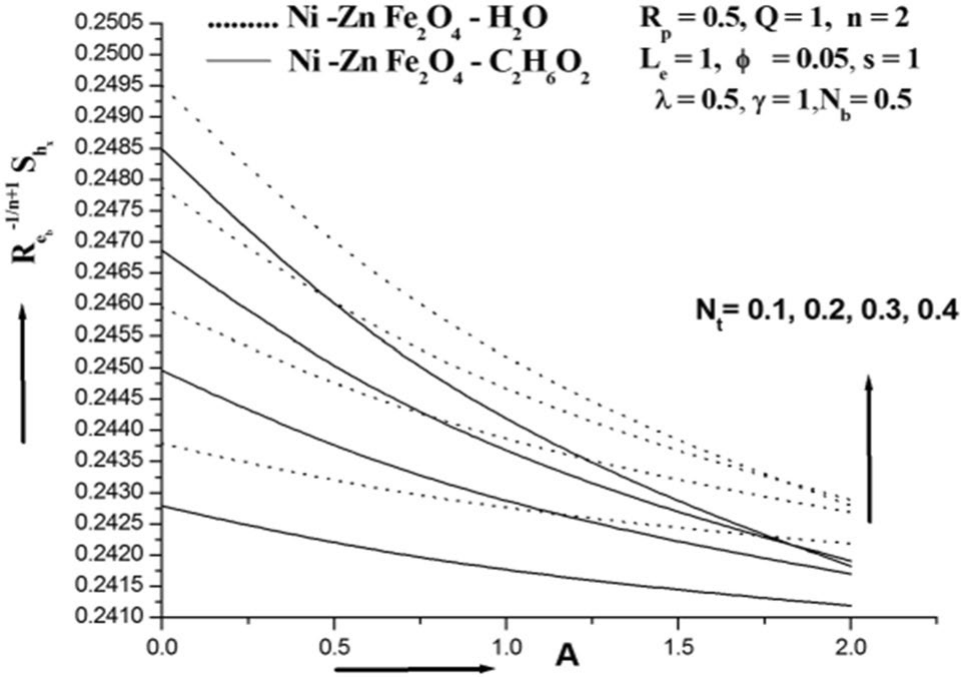
Sherwood number for various N_t_.

**Figure 8:**
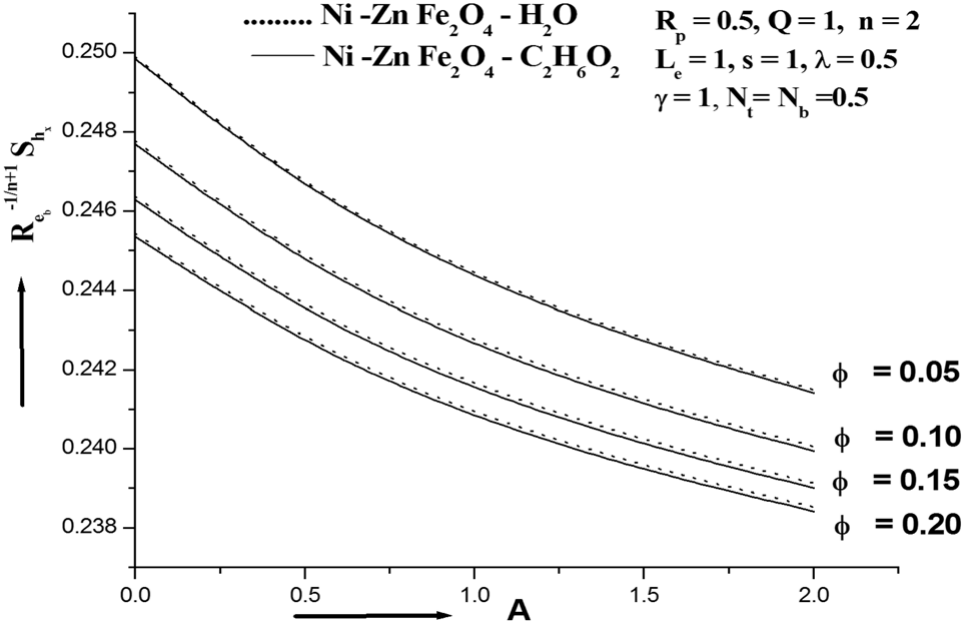
Sherwood number for various ϕ.

Figures 6, 7, 8 show the effect of the Brownian motion parameter Nb, the thermophoresis parameter (Nt), and the ferro nanoparticle solid volume fraction (ϕ) on the Sherwood number is shown in Figs. 6, 7, 8. Thermotransport is a crucial step in increasing Ferrofluid's thermal conductivity and is facilitated by the Brownian motion of ferroparticles. It is clear that a rise in the Brownian motion parameter Nb is the cause of the mass transfer rate's drop. The above results have a physical explanation that involves an improvement in fluid intermolecular collisions, increased Nb and decreased Sherwood number.

Figure 7 shows how the thermophoresis parameter Nt affects the ferro nanoparticle Sherwood number. The Sherwood number's profile is immediately raised by the increase in Nt. Physically, the higher mass flux that significantly raises the Sherwood number is associated to the higher value of Nt. Figure 8 demonstrates that the Sherwood number profile declines as a result of increasing values. This is because more nanoparticles cause fluid friction, which slows the flow as their volume increases. This illustration shows how this agreement and physical behaviour are related.When the Sherwood number falls,the volume fraction of nanoparticles does as well. It is well known that when Nt increases in value, the Sherwood number also rises. However, the Nb and ϕ affects both lower the Sherwood number. Mn–Zn Fe_2_O_4_–H_2_O and CoFe_2_O_4_–H_2_O are found to have lower and higher Sherwood numbers than Mn–Zn Fe_2_O_4_–C_2_H_6_O_2_ and CoFe_2_O_4_–C_2_H_6_O_2_, respectively.

For particular values of Nb, Nt, M Rp, and A, Table 3 shows the Sherwood number of the Siskoferronanofluids Mn–Zn–Fe_2_O_4_–H_2_O, Mn–Zn Fe_2_O_4_–C_2_H_6_O_2_, CoFe_2_O_4_–H_2_O, and CoFe_2_O_4_–C_2_H_6_O_2_. This table shows that the Sherwood number rises with M and Nt and falls with A, Rp, and Nb. Mn–Fe_2_O_4_–H_2_O and CoFe_2_O_4_–H_2_O are found to have lower and higher Sherwood numbers than Mn–Fe_2_O_4_–C_2_H_6_O_2_ and CoFe_2_O_4_–C_2_H_6_O_2_, respectively.

**Table 3:** Sherwood number of various Siskoferronanofluids.

	Cobalt ferrite (CoFe_2_O_4_)					Manganese Zinc ferrite				
**Water-based ferrofluid**										
**ϕ**	**N**_**t**_** = 0.2**	**N**_**b**_** = 0.2**	**M = 1.0**	**R**_**p**_** = 0.5**	**A = 1**	**N**_**t**_** = 0.2**	**N**_**b**_** = 0.2**	**M = 1.0**	**R**_**p**_** = 0.5**	**A = 1**
0.05	0.24466	0.27254	0.24900	0.24900	0.24900	0.24461	0.27208	0.24888	0.24888	0.24888
0.15	0.24231	0.25344	0.24359	0.24359	0.24359	0.24224	0.25294	0.24345	0.24345	0.24345

**ϕ**	**N**_**t**_** = 0.5**	**N**_**b**_** = 0.5**	**M = 2.0**	**R**_**p**_** = 1.0**	**A = 2**	**N**_**t**_** = 0.5**	**N**_**b**_** = 0.5**	**M = 2.0**	**R**_**p**_** = 1.0**	**A = 2**
0.05	0.249	0.249	0.24915	0.24884	0.24819	0.24888	0.24888	0.24904	0.24873	0.24810
0.15	0.24359	0.24359	0.24366	0.2435	0.24313	0.24345	0.24345	0.24352	0.24336	0.24301

**Ethylene glycol-based ferrofluid**										
ϕ	N_t_ = 0.2	N_b_ = 0.2	M = 1.0	R_p_ = 0.5	A = 1	N_t_ = 0.2	N_b_ = 0.2	M = 1.0	R_p_ = 0.5	A = 1
0.05	0.24462	0.27216	0.2489	0.2489	0.2489	0.24485	0.27238	0.24895	0.24895	0.24895
0.15	0.24226	0.25309	0.24349	0.24349	0.24349	0.24243	0.25335	0.24356	0.24356	0.24356

Φ	N_t_ = 0.5	N_b_ = 0.5	M = 2.0	R_p_ = 1.0	A = 2	N_t_ = 0.5	N_b_ = 0.5	M = 2.0	R_p_ = 1.0	A = 2
0.05	0.2489	0.2489	0.24905	0.24875	0.24813	0.24895	0.24895	0.2491	0.2488	0.24811
0.15	0.24349	0.24349	0.24355	0.24341	0.24307	0.24356	0.24356	0.24363	0.24348	0.24303

## Conclusion

The characteristics of variables influencing the mechanisms of concern are used to forecast action in a specific physical state. The assumption might be whether any of these amounts will affect the outcome of the change effort in geometrical, mass flow, flow characteristics, and so on. The primary goal of this research is to develop a mathematical framework of assumptions for mass transfer, fluid flow, and related operations. In short, of all estimation strategies, the mathematical approach has the most intention.

The effectiveness of Sisko ferro nanofluid mass transfer using the ferro particles manganese zinc ferrite (Mn1/2Zn1/2Fe_2_O_4_), cobalt ferrite (CoFe_2_O_4_) and nickel zinc ferrite (Ni–Zn Fe2O_4_) with water (H_2_O) and ethylene glycol (C_2_H_6_O_2_) as the base fluid on a porous surface is examined. The effects of parameters related to concentration and Sherwood number are illustrated using figures and tables. Following is a summary of this study.

1. The fluid's concentration rises with an increase in M and γ and falls with a reduction in A and Rp.
2. The Sisko ferro nanofluids' Sherwood number rises with M and N_t_ and falls with ϕ, A, Rp, Nb.
3. Siskoferronanofluid based on ethylene glycol has a lower mass transfer rate than that of water-based Siskoferronanofluid containing ferro particles nickel–zinc ferrite (Ni–ZnFe_2_O_4_) and cobalt ferrite (CoFe_2_O_4_), manganese–zinc. Ferrite (Mn1/2Zn1/2Fe_2_O_4_).

Current analyzes are directly relevant to next-generation mass transfer technology, vertical surface material processing, the chemical industry and all processes strongly influenced by mass transfer principles. This research enabled engineers to understand the most important processes in chemical processes. The use of mass transfer fluids incorporating ferro particle suspensions in Siskofluids to resolve the cooling issue in thermal systems is one scientific application of ferro particles with enormous potential. As a result, the coexistence of Brownian motion and thermophoretic subatomic condensation on magnetic field sisko ferrofluids is critical for both basic and applied sciences worldwide.

## Data Availability

The datasets used and analyzed during the current study are available from the corresponding author on request.

## List of symbols

$K_{p}$: Permeability of the medium (m^2^)
Q’: Volumetric rate of heat absorption
T: Temperature of the fluid (K)
$T_{\infty }$: Temperature away from the surface
${\text{D}}_{{\text{B}}}$: Brownian diffusion coefficient (m^2^/s)
${\text{D}}_{{\text{T}}}$: Thermophoresis diffusion coefficient (m^2^/s)
E_0,1_: Positive constant
ϕ: Solid volume nanoparticle fraction (mol/m^3^)
k_nf_: Thermal conductivity of nanofluid (W/mK)
k_f_: Thermal conductivity of base fluid (W/mK)
k_p_: Thermal conductivity of nanoparticle (W/mK)
μ_nf_: Dynamic viscosity of nanofluid (Ns/m^2^)
μ_f_: Dynamic viscosity of base fluid (Ns/m^2^)
μ_p_: Dynamic viscosity of nanoparticle(Ns/m^2^)
ρ_nf_: Density of nanofluid (kg/m^3^)
ρf: Density of base fluid(kg/m^3^)
ρ_p_: Density of nanoparticle(kg/m^3^)
ν_nf_: Kinematic viscosity of nanoparticle (m^2^/s)
ν_f_: Kinematic viscosity of base fluid(m^2^/s)
ν_p_: Kinematic viscosity of nanoparticle(m^2^/s)
(C_P_)nf: Specific capacity of nanofluid (J/kg K)
(C_P_)f: Specific capacity of base fluid(J/kg K)
(C_P_)p: Specific capacity of nanoparticle(J/kg K)
a: Viscosity parameter
m&n: Power low index
b: Consistency index
P: Pressure
I: The identity tensor
A_1_: The kinematic tensor
S: Extra stress tensor
τ: Cauchy stress tensor
